# Conflicts of Interest in Medicine. A Systematic Review of Published and Scientifically evaluated Curricula

**DOI:** 10.3205/zma001114

**Published:** 2017-08-15

**Authors:** Janosch Weißkircher, Cora Koch, Nadine Dreimüller, Klaus Lieb

**Affiliations:** 1 Universitätsmedizin der Johannes Gutenberg-Universität Mainz, Körperschaft des öffentlichen Rechts, Klinik für Psychiatrie und Psychotherapie, Mainz, Deutschland

**Keywords:** Medical Education, Curriculum, Medical/Psychology Students, Drug Industry, Advertising as Topic, Conflict of Interest

## Abstract

**Objective: **Conflicts of interests resulting from interactions with pharmaceutical companies are pervasive in medicine and can result in an undue influence on physicians’ decision-making. The objective of this systematic review is to analyze published and scientifically evaluated curricula for medical students and residents regarding such conflicts of interest. We begin by describing the covered topics and teaching methods; afterwards we analyze the quality of the curricula using the published data on their evaluations and comparing the content with content recommended for such curricula.

**Methods: **We searched Pubmed, PsycInfo, EMBASE, OECD, WISO, SOWI and googlescholar up to and including the 5th of September 2016. Publications describing curricula for residents or medical students on the topic of conflicts of interest in medicine and evaluating them for their effects on the participants’ learning were included. We analyzed the covered topics and the teaching methods used and compared them with recommendations by the American Medical Students’ Association (AMSA) and Health Action International (HAI).

**Results:** The literature search resulted in 20 publications that fulfilled our search criteria. In five trials, a control group was used, in no trial the participants were randomized to intervention or control group. 16/20 published curricula primarily covered marketing strategies by pharmaceutical companies, especially the interaction with pharmaceutical sales representatives (PSRs). Most curricula only covered a limited number of topics recommended by AMSA/HAI. The most frequent teaching method was a group discussion, which was used in 18/20 curricula; all curricula used at least one interactive teaching method. The evaluation of the curricula was heterogeneous in results as well as design. Some publications described a change of attitudes toward a stronger skepticism regarding interactions with pharmaceutical companies. Four publications described improved knowledge, one publication described a change in behavior toward a reduction of the acceptance of gifts.

**Conclusion: **The trials conducted to this date regarding curricula on conflicts of interests are methodologically flawed and the described curricula lack important topics beyond marketing strategies of pharmaceutical companies. In addition, there are no data so far on the sustainability of the courses’ effects on participants’ behavior. It is therefore necessary to develop a model curriculum that covers a broader variety of topics and to evaluate it using a well thought-out methodology to create a foundation for the further improvement of teaching conflicts of interest in medicine.

## Zusammenfassung

**Zielsetzung:** Interessenkonflikte durch Kontakte mit pharmazeutischen Unternehmen (pU) sind in der Medizin häufig und können zu einer unangemessenen Beeinflussung von ärztlichen Entscheidungen führen. Ziel dieser Übersichtsarbeit ist es, publizierte und wissenschaftlich evaluierte Lehrangebote für Studierende oder Assistenzärzte zu solchen Interessenkonflikten zu analysieren. Dabei werden zunächst die behandelten Themenkomplexe und angewandten Lehrmethoden dargestellt und im Anschluss die Qualität der Curricula anhand der publizierten Evaluationsdaten sowie des Abgleichs mit empfohlenen Themenschwerpunkten analysiert.

**Methodik:** Im Rahmen der Literatursuche wurden die Datenbanken Pubmed, PsycInfo, EMBASE, OECD, WISO, SOWI und googlescholar bis einschließlich 05.09.2016 durchsucht. Eingeschlossen wurden Publikationen, die Curricula mit Assistenzärzten oder Medizinstudierenden zum Thema Interessenkonflikte in der Medizin beschreiben und diese ggf. in Hinblick auf die erzielten Lerneffekte evaluieren. Die behandelten Themen und Lehrmethoden sowie die Methodik und die Ergebnisse der Evaluationen wurden analysiert und mit Empfehlungen der American Medical Students‘ Association (AMSA) und Health Action International (HAI) verglichen.

**Ergebnisse:** Die Literatursuche ergab 20 Publikationen, die den Kriterien der Suche entsprachen. Fünf Studien setzten eine Kontrollgruppe ein, keine Studie randomisierte die Teilnehmer auf die Interventions- und Kontrollgruppe. Thematisch behandeln 16/20 der publizierten Curricula vorwiegend Marketingstrategien von pU, insbesondere die Interaktion mit Pharmavertretern (PV). Die meisten Curricula deckten einen geringen Teil der von AMSA/ HAI empfohlenen Themengebiete ab. Als häufigste Lehrmethode wurden bei 18/20 Curricula Gruppendiskussionen eingesetzt; alle nutzten mindestens eine interaktive Lehrmethode. Die Evaluation der Curricula war in der Durchführung und den Ergebnissen sehr heterogen. Es wurden jedoch in einigen Publikationen Einstellungsänderungen der Teilnehmenden hin zu einem stärkeren Skeptizismus gegenüber Interaktionen mit pU berichtet. Vier Publikationen berichten von einem Wissenszuwachs, in einer Publikation wurde eine Verhaltensänderung der Teilnehmenden im Sinne einer Reduktion der Annahme von Geschenken beschrieben.

**Schlussfolgerung: **Bisher existierende Studien zu Lehrveranstaltungen zu Interessenkonflikten weisen methodische Mängel auf und die beschriebenen Curricula sparen wichtige Themen abseits von Marketingstrategien der pU aus. Zudem lässt sich bisher keine Aussage über nachhaltige Auswirkungen von Lehrveranstaltungen auf Verhaltensänderungen der Teilnehmer treffen. Es wäre daher wünschenswert, ein thematisch breiteres Mustercurriculum zu entwickeln und dieses methodisch solide zu evaluieren, um eine Grundlage zur weiteren Verbesserung der Lehre zu Interessenkonflikten in der Medizin zu schaffen.

## 1. Introduction

Conflicts of interest (COI) are defined as a set of circumstances that creates a risk of professional judgment concerning a primary interest being unduly influenced by a secondary interest [1]. For doctors, such conflicts may arise from interactions with pharmaceutical companies, among others [2], [3], [4]. Several studies have shown that industrial interests may influence physicians’ decisions and may thus have negative effects for patients and the health care system [5], [6]. Recent surveys have shown that medical students also interact with pharmaceutical companies on a regular basis and feel inadequately prepared for these interactions by their universities [4], [7], [8]. It therefore seems to be reasonable to raise awareness for the effects of COI among medical students and residents early on in their training.

Two associations have compiled and published suggestions for curricula regarding COI. The American Medical Students‘ Association (AMSA) has created a “model curriculum” based on recommendations by the Institutes of Medicine (IOM) as well as the American Association of Medical Colleges (AAMC) [9]. Health Action International (HAI), a non-governmental organization, has also prepared a similar manual in cooperation with the WHO [10]. To the knowledge of the authors, neither curriculum has been put into practice and evaluated in its entirety, but both represent fundamental recommendations for creating curricula on COI. 

So far, four reviews have studied the literature on COI; the most recent was published in 2008 [2], [11], [12], [13]. Because these reviews are either limited to students or to residents and because new courses have been published since the last review was published, a current review is missing that could serve as a guide for universities wishing to establish courses on COI. In addition, no review has so far assessed the curricula for the quality of their contents. The objective of this review is therefore to firstly present the topics covered and the teaching methods used in the curricula. Subsequently, the quality of the curricula is assessed using data from the evaluations as well as – for the first time – assessing the content quality by comparing the covered topics with the recommendations by AMSA and HAI. 

## 2. Methods

Pubmed, PsycInfo, EMBASE, OECD, WISO, SOWI and googlescholar were searched for publications on curricula regarding COI for medical students and/or residents. Publications in German or English language describing a curriculum that was evaluated were included. 

For the search in pubmed, the mesh-terms „Education, Medical”, “Curriculum”, “students, Medical/psychology”, “Drug Industry”, “advertising as topic”, and “Conflict of interest” were identified as relevant from a literature search and supplemented by the limit “humans”. The detailed search string as well as the search strings for the other databases can be found in attachment 1 . The databases were last searched on Sept. 5^th^, 2016, so that the timeframe of publication is from Jan. 1^st^, 1960 to Sept. 5^th^, 2016. Further articles were included from the reference lists of the included articles. 

The references were screened for inclusion into the review by JW, first by title and abstract and then by full text. From the included publications, general information such as author, publication date and target group, information regarding the curriculum such as number of participants, duration, content and teaching methods, as well as information regarding the evaluation of the curriculum such as number of surveyed participants, study design, number of items in the questionnaire, outcomes and results of the evaluation were extracted. To extract the content of the curriculum, categories were created after a primary reading of the publications and the topics described in each publication were then sorted into these categories. The covered topics were then compared with the recommendations made by the manuals of AMSA and HAI.

## 3. Results

**Figure 1:**
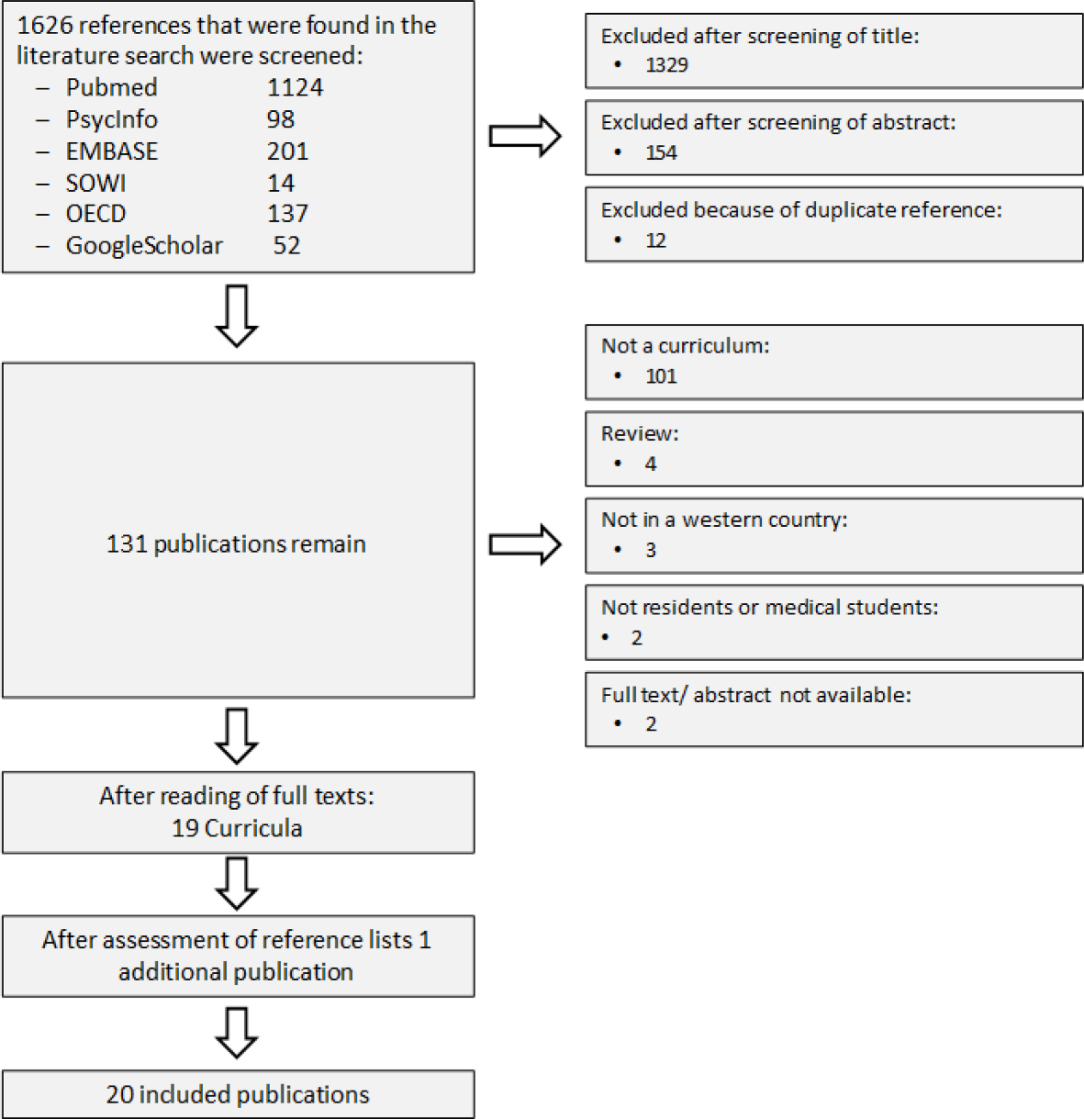
Flow-Chart – Systematic literature search

The primary literature search yielded 1,626 references. After screening of title, abstract and full text, 19 publications fulfilled the inclusion criteria. Figure 1 (Fig. 1) gives an overview of the reasons for exclusion. Two publications from Pakistan and one from Nepal were excluded from analysis because we assumed that the context of the health care system and the medical school curriculum are not comparable with those of western countries [14], [15], [16].

From the reference list of the 19 included publications, one additional publication was identified that fulfilled the inclusion criteria, so that 20 publications were included in the final analysis. Attachment 2 gives an overview of the characteristics of these 20 publications. 10 curricula were targeted toward medical students, 8 toward residents and 2 toward residents and faculty. The duration was between 40 min and 70 h, 8.16h on average (SD: 16.9h) and the median duration was 3h.

### 3.1. Topics covered by the curricula

Table 1 (Tab. 1) shows which topics were covered how often by each of the 20 curricula. Two publications did not give information regarding the covered topics [17], [18]. Five curricula cover a single topic each [19], [20], [21], [22], [23]. Eight curricula cover two topics each [19], [21], [22], [23], [24], [25], [26], [27]. Four curricula focus on three topics [28], [29], [24], [30].

#### 3.2. Comparison of the curricula with the recommendations by AMSA and HAI

Table 2 (Tab. 2) gives an overview of the topics recommended in the manuals by AMSA and HAI [9], [10], which manual recommends them and which curricula included in this review cover the recommended topics.

As is apparent from table 2 (Tab. 2), many curricula cover different marketing instruments used by pharmaceutical companies (16/20 curricula), their effect on clinical decision-making (15/20 curricula) and possibilities to handle these marketing instruments (16/20 curricula). Most curricula focus especially on the interaction with pharmaceutical sales representatives (PSR). 3/16 curricula that focus on marketing instruments also teach, which independent sources of information exist [28], [31], [32]. The other publications do not describe which types of strategies of handling the marketing instruments are taught; only one publication mentions that legal limits on interactions with PSR are taught [29]. 

COI resulting from interactions with pharmaceutical companies that are not marketing instruments are covered by two curricula. Two and one focus on research collaborations and non-interventional trials, respectively. No publication mentions covering sponsorship of educational events and conferences or conflicts of interests concerning clinical practice guidelines.

#### 3.3. Teaching methods used in the curricula

Table 3 (Tab. 3) gives an overview of the teaching methods used in the curricula. Presentations by instructors were used in 11 curricula. These additionally used interactive teaching methods. The methods used to prepare participants for the interaction with PSR were especially varied. In six curricula, videos of conversations with PSR were analyzed. In a further six curricula, role-playing was used, where participants trained interactions with PSR with each other or with an instructor or actual PSR. In two curricula, role-playing was organized as a cooperation with the pharmaceutical department at the university. In one, a pharmacist pretended to be a PSR [32]. In another, a PSR initially presented and following the PSR’s presentation, a pharmacist presented independent information on the same topic [30]. In both curricula, a critical discussion followed the presentation of the PSR. In another curriculum, a former PSR presented on the education of PSR, their sales strategies and strategies of interacting with PSR [23]. In six curricula, pharmaceutical companies were part of the development or teaching of the curriculum [19], [20], [29], [30], [26], [33].

#### 3.4. Analysis of the evaluation of the curricula

Table 4 (Tab. 4) summarizes the main characteristics of the evaluation of the curricula. All evaluations were exclusively based on questionnaires. 13 of 15 studies that used a pre- and posttest measured a change of attitude toward pharmaceutical companies as an outcome parameter. Five measured a knowledge gain [21], [29], [26], [34], [33]. Two asked for changes in behavior [18], [35]. In five studies, the intervention group was compared with a control group, but none of these studies employed randomization for the allocation to the groups [17], [23], [28], [24], [35]. Also in five studies, a follow up at least 3 months after the end of the course was conducted [28], [24], [36], [26], [32]. In one further study, it is not clear from the publication how much later the posttest was conducted [23].

The heterogeneity of the methods of evaluation makes a systematic presentation of the data difficult. Table 5 (Tab. 5) gives an overview; more detailed information with summaries of the results of each study can be found in attachment 3 . Following, the most important results regarding change in attitude, knowledge and behavior are presented. 

##### 3.4.1. Studies that studied changes in attitudes

Eight of twelve studies focusing on changes in attitudes in a pre- and posttest found a change toward more skeptical attitudes toward pharmaceutical companies [17], [18], [23], [28], [24], [36], [27], [32]. Three studies did not find a change in a single direction or found no change [29], [30], [35], one study found a change toward more positive attitudes toward pharmaceutical companies [33]. Mostly, the questionnaires assessed the perceived influence interactions with pharmaceutical companies have, the perception of the appropriateness of marketing-interactions as well as the perceived value of interactions with pharmaceutical companies. Following, the results of these studies are described, ordered by study design.

Uncontrolled studies that employed a posttest immediately after the intervention found heterogeneous results, see table 4 (Tab. 4) [18], [29], [30], [33]. 

Three of four controlled studies with a posttest immediately after the intervention found more skeptical attitudes of the participants after the intervention [17], [23], [27]. Vinson et al. (1993) asked for the willingness to accept gifts, which was significantly lower in the intervention group following the intervention for six of eleven gifts (p=0,03), while no changes were found in the control group [17]. Hopper et al. (1997) found a significant change in the agreement to three of eight statements regarding interactions with pharmaceutical companies toward a more critical attitude toward pharmaceutical companies [27]. The participants had a lower sense of the appropriateness of accepting gifts without value to the patient and thought that PSR interactions had a larger influence. Kao et al. (2011) also found that participants tended to recognize an influence of marketing practices more frequently. In addition, the intervention group agreed more often that interactions between pharmaceutical companies and students or doctors should be prohibited [23]. Randall et al. (2005) did not find significant differences in attitudes between pre- and posttest [35].

The two studies that did a posttest after more than three months but did not have a control group found that participants were more skeptical after the intervention [36], [32]. Shaughnessy et al. (1995) found a significant change toward a more skeptical attitude for three of ten items. The participants agreed more often that different marketing instruments influenced their prescribing practices. Other items on the questionnaire that did not show significant differences partly showed a trend in the opposite direction [36]. Wilkes and Hoffmann (2001) found a consistent trend toward a more skeptical attitude toward marketing strategies of pharmaceutical companies, which was significant for four of 26 items. Participants also rated the influence of marketing instruments higher and thought that sponsorship of educational events was less appropriate [32]. 

The two controlled studies that conducted a posttest after more than three months found more skeptical attitudes compared to before the intervention as well as compared to the control group [28], [24]. Daniel et al. (1966) asked for agreement regarding different statements about marketing by PSR and quality of different informational material and describe a significant difference that showed a more skeptical attitude of the intervention group. However, they do not report the size of the effect or the direction of change for each item (p<0.05) [28]. Schneider et al. (2006) asked to rate the appropriateness of 17 different interactions with pharmaceutical companies one year after participation in a workshop. One of the 17 items was rated as less appropriate by the intervention than by the control group (p=0.042) [24]. 

##### 3.4.2. Studies focusing on increase in knowledge

The five studies examining the knowledge of participants were not controlled [20], [21], [29], [26], [34]. In all cases except for the study by Anastasio & Little, knowledge was examined directly after the intervention [26]. One study did not conduct a pretest [20]. All studies describe an increase in knowledge after the intervention, in two cases this was based on a direct test of knowledge [34], [33] and in three cases on self-reports by participants [20], [21], [26]. Watkins & Kimberly (2004) describe an improvement in a multiple-choice exam from 53% to 88% (no p reported) [34]. Stanley et al. (2005) describe an average of 56.8% (standard error 3.3) on an exam after the intervention vs. 32.9% (standard error 3.7) before [33]. Tillmanns et al. (2007) found that the participants reported a significantly improved self-assessment of knowledge about interactions with pharmaceutical companies after the intervention [21]. Anastasio & Little (1996) found that residents felt more secure in their interactions with PSRs after the intervention [26]. This result was significant for ten of ten items. Kelcher et al. (1998), who did not conduct a pretest, described that participants felt better informed after the intervention [20].

##### 3.4.3. Studies that examined behavioral changes

In two studies, participants were asked for their behavior after participation in the curriculum; both were based on self-assessment [18], [35]. Agrawal et al. found that the intervention group reported lower intentions to use marketing instruments, but there was no significant difference when asked for the actual use of marketing instruments within the last month [18]. Randall et al. describe that the intervention group, as opposed to the control group, had reduced certain interactions with pharmaceutical companies, i.e. the acceptance of office stationery and other non-informative gifts. For five other interactions, no significant differences in the rates of acceptance were found [35].

## 4. Discussion

This is the first review that systematically analyzes and assesses the quality of all curricula regarding COI for residents and medical students published until September 2016. Regarding content, curricula tend to focus on marketing strategies of pharmaceutical companies, especially the interactions with PSR. Only few curricula name “conflicts of interest“ as an explicit topic, even though situations or interactions are described as being taught that represent COI according to the definition the authors of this review use. None of the curricula cover all the topics recommended by AMSA and HAI (see table 2 (Tab. 2)); all curricula are limited to one to three topics.

Regardless of the reasons for the limitations in covered content, this raises the question whether the curricula are sufficiently broad to actually improve the management of COI in practice. In the opinion of the authors, additionally teaching interactions that represent COI but are not strictly marketing practices as well as the critical interpretation of scientific data would be especially important. Both topics are only rarely covered by the curricula examined in this review.

The teaching methods that are used appear to be useful considering most curricula use at least two different methods and integrate at least one interactive teaching method [37]. The emphasis on interactions with PSRs by most authors is obvious here as well, as this topic was taught using a very broad variety of teaching methods. However, usually the teaching of alternatives to PSRs was missing, as was already stated by Montague [12].

In six curricula, employees of pharmaceutical companies were directly involved, which the authors of this review view critically. Young doctors should be prepared for interactions with pharmaceutical companies because it is likely they will interact with pharmaceutical companies during their professional life. However, these interactions can be taught or practiced without the involvement of PSRs, as several curricula showed [21], [23], [25], [32]. Furthermore, the involvement of pharmaceutical companies bears the risk that contents of the curriculum are unduly influenced by the interests of the involved company, as has been noted by AMSA and Montague [9], [12]. One hint that this is actually the case is that the only changes toward a more positive attitude toward pharmaceutical companies were found in curricula where pharmaceutical companies were involved [29], [33].

Based on the included publications, only very limited conclusions regarding the effects on learning of participants can be drawn, as has been stated in previous reviews on the topic [2], [11], [12], [13]. This is due to methodological flaws in the evaluation of effects in many studies on the one hand (i.e. missing control group, no randomization). On the other, important information is missing from several publications that could serve to better assess the achieved effects. In addition, the heterogeneity of studies further complicates a comparison. However, it can be said that the studied curricula led to changes in attitude, though only small and partly conflicting effects could be shown. In four studies, of which two were controlled, a sustainable change in attitude toward a more skeptical attitude toward pharmaceutical companies was found even several months after the participation in the curriculum [28], [24], [36], [32]. Only two studies without a control group seem to show a knowledge gain after the intervention [34], [33]. However, in one publication information regarding what was asked for and the p value is missing, which complicates the interpretation of results [34]. Only one of two studies that examined behavioral change found such a change of behavior toward a reduced frequency of interactions with industry. In this case, information regarding absolute changes and absolute frequency of interactions were missing, which could have served to assess the effect sizes [35].

## 5. Conclusions

To summarize, the published curricula give indications that curricula can be effective in inducing changes in attitude in young doctors. Whether these lead to sustainable behavioral changes has so far not been studied in enough depth. Clear recommendations as to which building blocks should be part of a model curriculum cannot be made based on the included studies. For faculties aiming to establish a course on COI or management of interactions with pharmaceutical companies, guidance is thus lacking. Therefore, it seems necessary to conduct methodologically sound studies on this topic, that assess immediate effects on learning as well as and especially long-term effects. This could help to improve teaching in medicine on COI and the management of interactions with pharmaceutical companies by medical students and doctors. 

## Funding

Funded by Volkswagen Foundation

## Competing interests

The authors declare that they have no competing interests.

## Supplementary Material

Search strings

Overview of the included curricula, sorted by year of publication

Results of the evaluation of the curricula, sorted by study design

## Figures and Tables

**Table 1:**
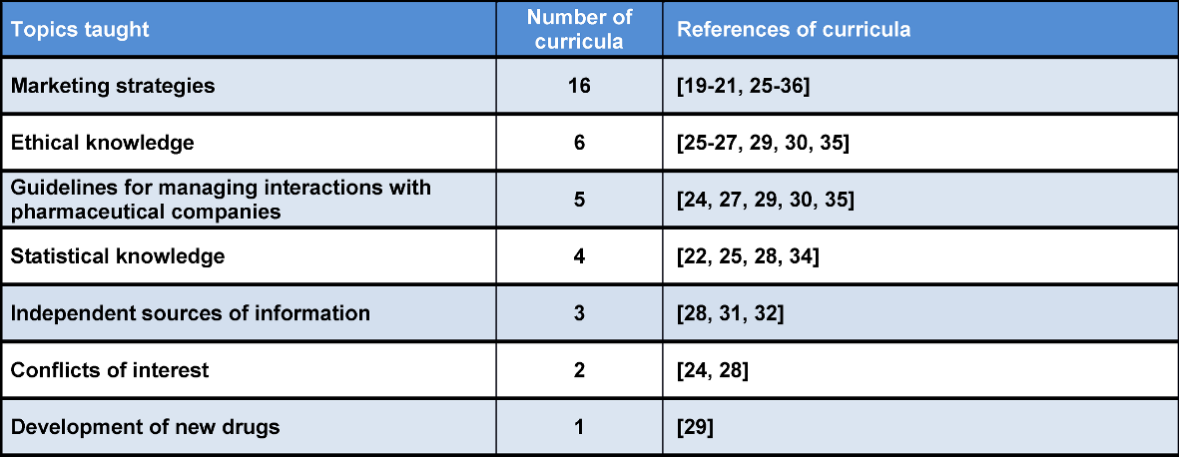
Topics taught in the curricula, sorted by frequency

**Table 2:**
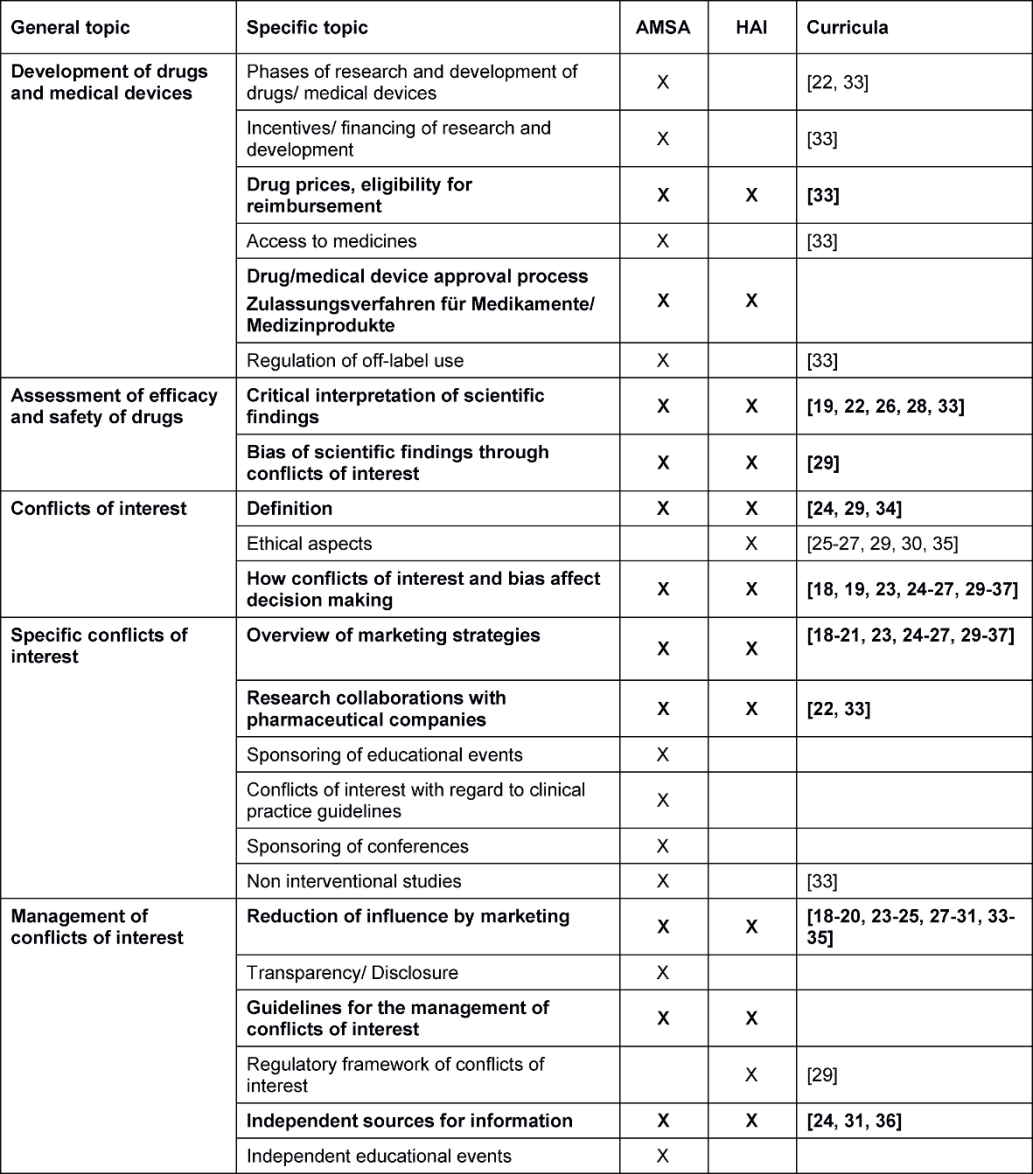
Topics recommended by AMSA and HAI as well as implementation in the included curricula. Topics recommended by both manuals are highlighted in bold.

**Table 3:**
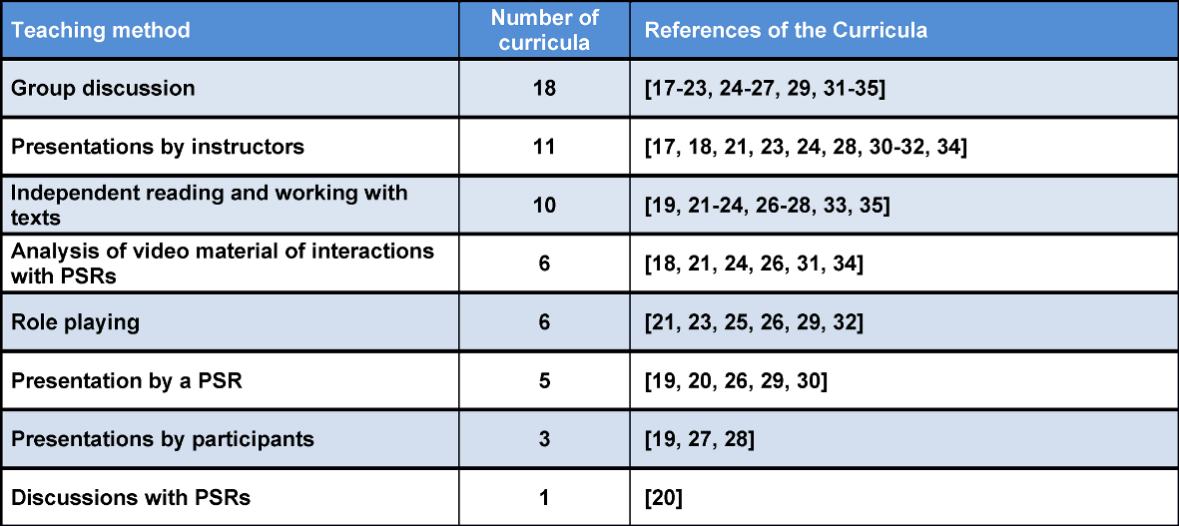
Teaching methods used, sorted by frequency of use

**Table 4:**
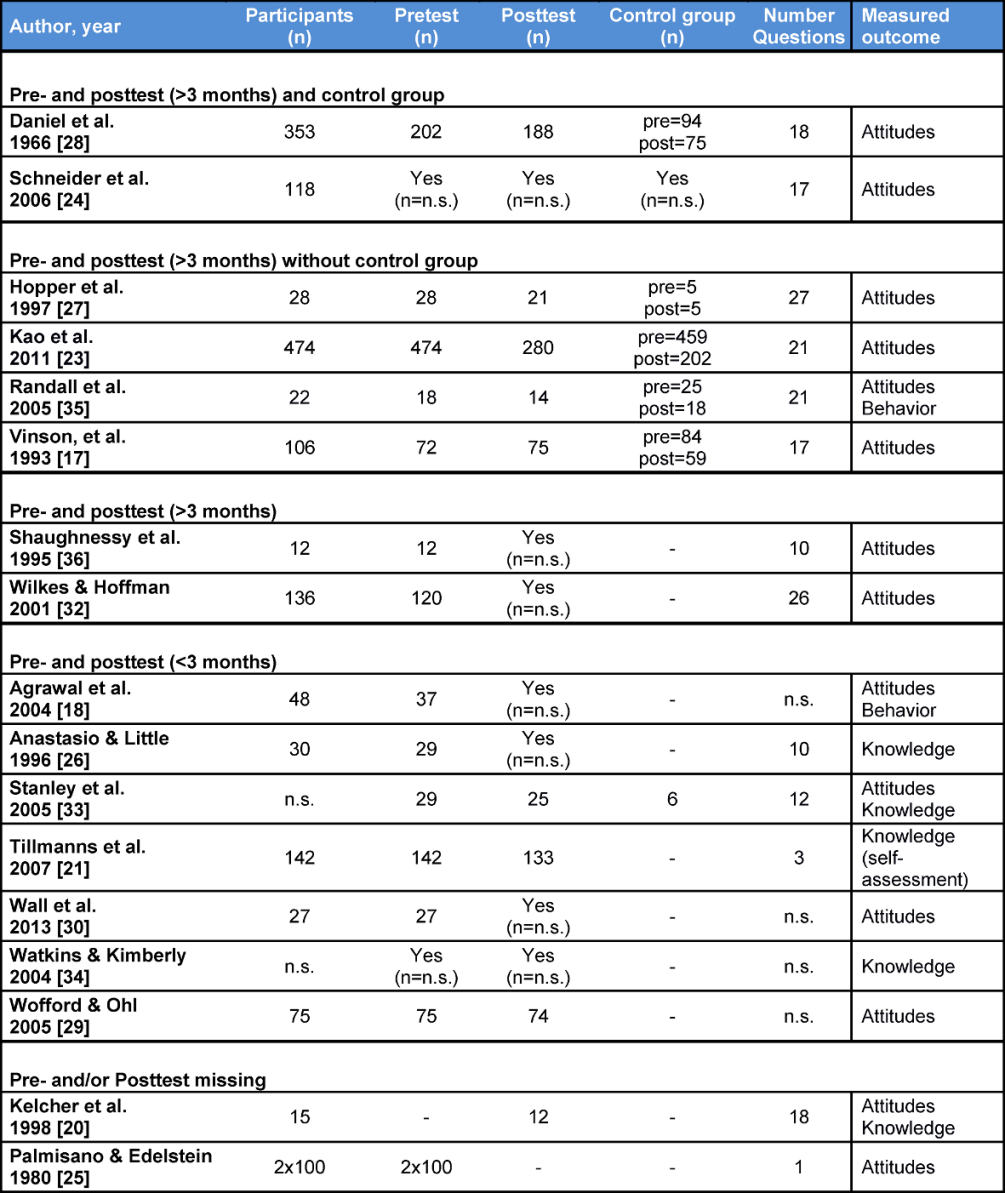
Overview of the evaluations of the curricula, sorted by study design (n=number of participants, n.s.=not specified; three publications are not included in this table because there was no information on the evaluation [19, 22, 31]).

**Table 5:**
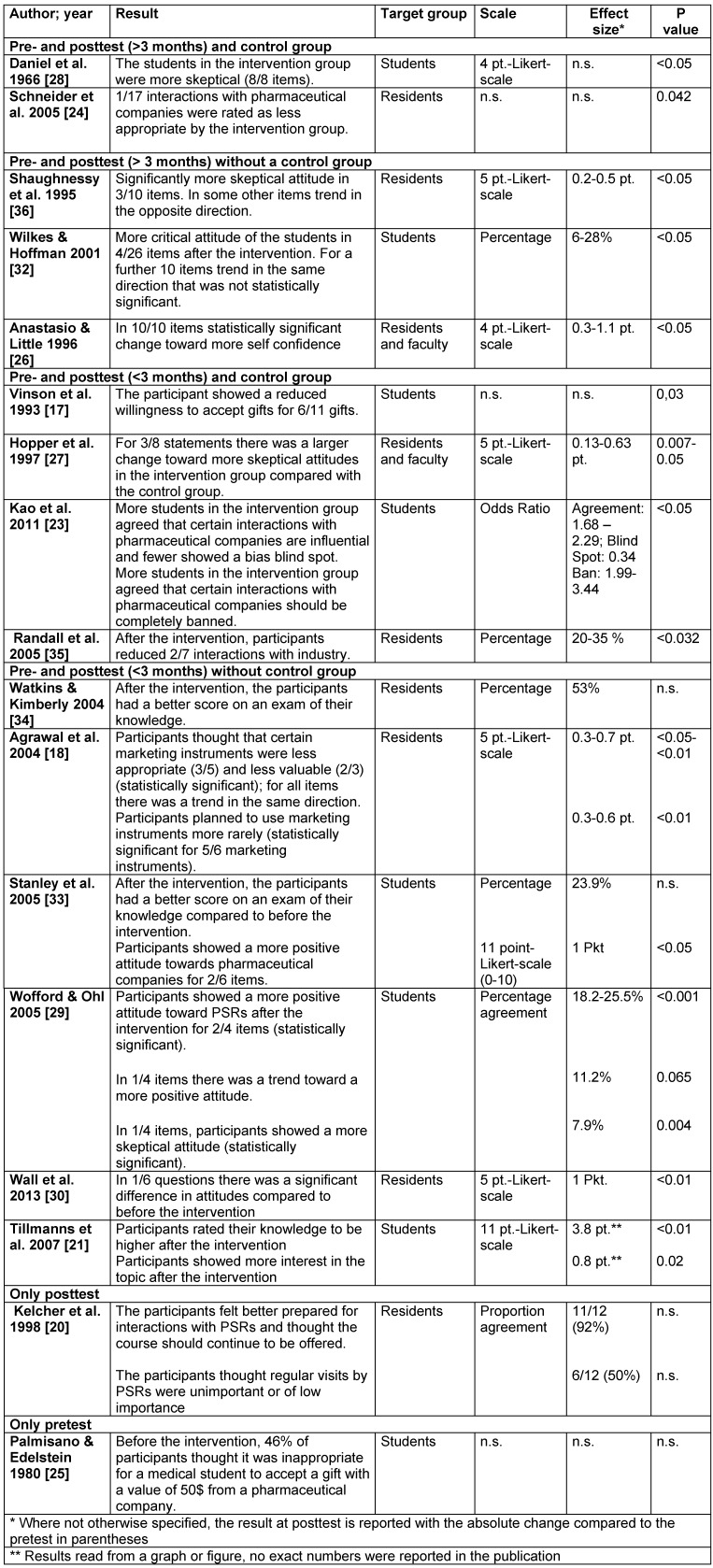
Results of evaluation of curricula, sorted by study design (abridged)
