# Supplementary material for: Conflicts of Interest in Medicine. A Systematic Review of Published and Scientifically evaluated Curricula
Source: GMS J Med Educ. 2017 Aug 15;34(3):Doc37. doi: 10.3205/zma001114 (PMC5569982; doi:10.3205/zma001114)
Supplement: Search strings [file JME-34-37-s-001.pdf]

German terms within search strings are translated in curly brackets where the whole search string is not translated

Search string for Pubmed:
("Education, Medical"[Mesh] OR "Curriculum"[Mesh] OR "Curriculum" [ALL Fields] OR "education"[title] OR "educational"[title] OR "Students, Medical/psychology"[MESH]) AND ("Drug Industry"[Mesh] OR "industry"[title] OR "advertising as topic"[Mesh] OR "Conflict of Interest"[Mesh]) AND "humans"[MeSH Terms] AND (ger[LA] OR eng[LA])

Search string for PsycInfo, EMBASE:

- (FT=Conflict of Interest AND FT=Education) AND (LA=ENGLISH OR LA=GERMAN) AND pps=Mensch {human}
- (FT=Interessenkonflikt AND FT=Ausbildung) AND (LA=ENGLISH OR LA=GERMAN) AND pps=Mensch {human}

Search string for SOWI:

- (Alle Felder: Conflict of Interest) AND (Alle Felder: Education) AND (Sprache:"Englisch (EN)") OR (Sprache:"Deutsch (DE)")
  - Translation: (all fields: conflict of interest) AND (all fields: education) AND (language:"english (EN)") OR (language:"German(DE)")
- (Alle Felder: Interessenkonflikt) AND (Alle Felder: Ausbildung) AND (Sprache:"Englisch (EN)") OR (Sprache:"Deutsch (DE)")
  - Translation: (all fields: conflict of interest) AND (all fields: education) AND (language:"english (EN)") OR (language:"German(DE)")

In variable combinations:

- Pharmaindustrie, Pharmaunternehmen, Interessenkonflikt, Ausbildung, Studierende, Medizin, Assistenzärzte, Curriculum
  - Translation: Pharmaceutical industry, pharmaceutical company, conflict of interest, education, students, medicine, residents, curriculum

OECD:

- Search with the terms: Interessenkonflikt {conflict of interest}, Studierende {students}, Medizin {medicine}, Pharmaindustrie {pharmaceutical industry}, Pharmaunternehmen {pharmaceutical company}, Conflict of Interest, Students, Education, industry; Restricted to Language(s) German AND English; Published Between 1900 and 2017

WISO:

- Interessenkonflikt Medizin Studierende …

  … Ausbildung
  … Assistenzärzte
  … Pharmaindustrie
  - Translation: Conflict of interest medicine students …
    - … education
    - … residents
    - … pharmaceutical industry

GoogleScholar:

- allintitle: Conflict of Interest education
