# Supplementary material for: Conflicts of Interest in Medicine. A Systematic Review of Published and Scientifically evaluated Curricula
Source: GMS J Med Educ. 2017 Aug 15;34(3):Doc37. doi: 10.3205/zma001114 (PMC5569982; doi:10.3205/zma001114)
Supplement: Overview of the included curricula, sorted by year of publication [file JME-34-37-s-002.pdf]

Author	Year	Country	Target group	Participants (n)	Duration (in hours)
**Garb [19]**	1960	USA	Students	60	11
**Daniel et al. [28]**	1966	Canada	Students	n.s.	n.s.
**Palmisano & Edelstein [25]**	1980	USA	Students	2x100	1,5
**Vinson, et al. [17]**	1993	USA	Students	84	50 min
**Shaughnessy et al. [36]**	1995	USA	Residents	12	n.s.
**Anastasio & Little [26]**	1996	USA	Residents and Faculty	30	3
**Shear et al. [31]**	1996	Canada & Australia	Students	n.s.	1
**Hopper et al. [27]**	1997	USA	Residents and Faculty	49	40 min
**Kelcher et al. [20]**	1998	Canada	Residents	15	6
**Wilkes & Hoffman [32]**	2001	USA	Students	136	4
**Agrawal et al. [18]**	2004	Canada	Residents	48	2,5
**Watkins & Kimberly [34]**	2004	USA	Residents	n.s.	4
**Randall et al. [35]**	2005	USA	Residents	22	1,5
**Stanley et al. [33]**	2005	UK	Students	n.s.	70
**Wofford, Ohl [29]**	2005	USA	Students	75	1,5
**Schneider et al. [24]**	2006	USA	Residents	118	n.s.
**Tillmanns et al. [21]**	2007	USA & Germany	Students	142	15
**Merrill et al. [22]**	2010	USA	Residents	n.s.	n.s.
**Kao et al. [23]**	2011	USA	Students	474	3
**Wall et al. [30]**	2013	USA	Residents	27	5
