# Supplementary material for: Conflicts of Interest in Medicine. A Systematic Review of Published and Scientifically evaluated Curricula
Source: GMS J Med Educ. 2017 Aug 15;34(3):Doc37. doi: 10.3205/zma001114 (PMC5569982; doi:10.3205/zma001114)
Supplement: Results of the evaluation of the curricula, sorted by study design [file JME-34-37-s-003.pdf]

Author; year	Content of Questionnaire	Results	Scale / effect size / statistical significance
**Pre- and posttest (>3 months) and control group**			
**Daniel et al. 1966 [28]**	Agreement to statements regarding pharmaceutical marketing	The students in the intervention group were more skeptical (8/8 items).<br>• Drug companies are not accurate in their claims for their products.<br>• Drug companies do not induce physicians to increase the cost of therapy by using new drugs when equally effective older remedies are available.<br>• The claims made for drugs in mailed literature are not accurate.<br>• The price of therapy when new drugs are used is unnecessarily high because of the existence of equally effective, older, cheaper remedies.<br>• Information from detail men regarding claims about drugs is accurate.<br>• Drugs are not placed on the market before being adequately tested.<br>• Physicians are persuaded by advertising to use new drugs before they have been adequately tested.<br>• Drug companies do not try to be accurate in their claims for their products.	4-pt.-Likert-scale / size or direction of change not specified / p<0.05
**Schneider et al. 2005 [24]**	Appropriateness of different interactions with pharmaceutical companies	1/17 items were rated as less appropriate by the intervention group.<br>• Sponsored lunch	Scale and size not specified / p=0.042
**Pre- and posttest (>3 months) without control group**			
**Shaughnessy et al. 1995 [36]**	Agreement to statements regarding interactions with pharmaceutical companies	3/10 items with a significant change toward more skeptical attitudes. In some other items, trend in the opposite direction.<br>• Discussion with PRs has no impact on my prescribing behavior.<br>• Acceptance of promotional items from PRs has no impact on my prescribing behavior.<br>• PRs help to support important conferences and speakers at this institution.	Average on a 5-pt.-Likert-scale, 1=strongly agree<br>• 3.3 (+0.2, p<0.05)<br>• 2.3 (+0.5, p<0.05)<br>• 2.2 (+0.5, p<0.05)
**Wilkes & Hoffman 2001 [32]**	Agreement to statements about interactions between doctors and pharmaceutical companies<br><br>Agreement to statements about ethical aspects of interactions with pharmaceutical companies	In 4/26 items, the students showed a more critical attitude after the intervention.<br>• When drug companies sponsor physicians to go to seminars at resort locations this biases the subsequent behavior of those physicians (e.g., they prescribe more of the company's product).<br>• When drug companies give physicians pens, calendars, or other non-educational materials, this biases the subsequent behavior of those physicians.<br>• Product information presented in a drug advertisement provides you with educational material about the drug.<br><br>• It is unethical for physicians to accept drug company funding to attend seminars at resort locations.<br><br>For 10 other items, there was a trend in the same direction that was not statistically significant.	Percentage of participants that agreed<br>• 46% (+28%, p<0.05)<br>• 20% (+ 7%, p<0.05)<br>• 43% (-6%, p<0.01)<br><br>• 33% (+ 7%, p<0.05)

**Anastasio & Little 1996 [26]**	Confidence in interactions with pharmaceutical sales representatives	In 10/10 items a statistically significant change toward more self confidence<br>• Time management<br>• Control of the agenda<br>• Analyzing research results<br>• Giving feedback<br>• Identifiying marketing techniques<br>• Managing marketing techniques<br>• Managing the acceptance of gifts<br>• Asking for information<br>• Asking for drug samples<br>• Getting useful information	Average on a 4-pt.-Likert-scale, 4= very self-confident**<br>• 3.1 (+0.7, p<0.05)<br>• 3.2 (+1, p<0.05)<br>• 2.7 (+0.6, p<0.05)<br>• 3.1 (+0.8, p<0.05)<br>• 3.3 (+1.1, p<0.05)<br>• 3.2 (+1.1, p<0.05)<br>• 3.2 (+0.3, p<0.05)<br>• 3.4 (+0.7, p<0.05)<br>• 3.2 (+0.5, p<0.05)<br>• 3.3 (+0.7, p<0.05)
**Pre- and posttest (<3 months) and control group**			
**Vinson et al. 1993 [17]**	Willingness to accept gifts	The participants showed a lower willingness to accept gifts for 6/11 gifts.<br>• Medical textbook<br>• Promotional brochure<br>• Medical journal that is solely funded through advertising<br>• Pen<br>• Evening educational event<br>• Travel costs to a scientific event in a resort hotel	size not specified / p=0.03
**Hopper et al. 1997 [27]**	Attitudes toward different interactions with pharmaceutical companies	For 3/8 statements there was a change in attitude toward a more skeptical attitude of the intervention compared to the control group.<br>• Interactions with PRs are likely to influence the prescribing behavior of other physicians in negative ways<br>• PRs may use unethical marketing practices<br>• It is ethically appropriate to receive marketing gifts without patient benefit	Change on a 5 pt.-Likert-scale; 5=strong agreement<br>• 0.13 (control: -0.4); p=0.046<br>• 0.63 (control: -0.2); p=0.007<br>• -0.37 (control: 0.24); p=0.050
**Kao et al. 2011 [23]**	Perceived influence of marketing	More students in the intervention group agreed that certain interactions are influential and fewer students showed a bias blind spot.<br>• Receiving gifts or food from a pharmaceutical representative increases the chance I will eventually prescribe the company's drug.<br>• Marketing or promotional activities have a moderate or significant influence on physician prescribing decisions<br>• Food/gifts do not influence my own prescribing decisions, but those of my fellow medical students.	Percentage of participants that agreed<br>• 55.4% (OR 1.68 vs. control group)<br>• 72.2% (OR 2.29 vs. control group)<br>• 5.9% (OR 0.34 vs. control group)
	Attitude toward a ban of interactions with pharmaceutical companies	More students in the intervention group agreed, that certain interactions should be completely banned:<br>• Pharmaceutical sales representatives - doctors<br>• Pharmaceutical sales representatives - medical students	• 51.9% (OR 3.44 vs. control group)<br>• 57.1% (OR 1.99 vs. control group)

**Randall et al. 2005 [35]**	Agreement with statements regarding interactions with pharmaceutical companies	No difference	• -
	Acceptance of gifts (self-report)	After the intervention, the participants reduced 2 of 7 interactions with pharmaceutical companies<br>• Miscellaneous office supplies<br>• Non-educational gifts	Reduction compared to baseline<br>• 35% (F=17.28, p=0.0001)<br>• 20% (F=4.83, p=0.032)
**Pre- and posttest (<3 months) without control group**			
**Watkins & Kimberly 2004 [34]**	Multiple Choice Test; content not specified	The participants had a better score after the intervention	Percentage of correct answers / 86% (+53%) / p not specified
**Agrawal et al. 2004 [18]**	Ethical appropriateness and value of different marketing instruments	The participants rated certain marketing instruments as less appropriate (3/5) and less valuable (2/3) (statistically significant); there was a trend in the same direction for all items.<br>Ethical appropriateness in general<br>• Drug samples<br>• Free meals<br>• Gift less than CAN $10<br>Value in general<br>• Drug sample<br>• Industry-sponsored continuing medical education	Averages on a 5-pt.-Likert-scale (5=very appropriate or very valuable)<br>n.s., p<0.05<br>• 3.4 (-0.5, p<0.01)<br>• 2.3 (-0.3, p<0.01)<br>• 2.1 (-0.7, p<0.01)<br>n.s., p<0.05<br>• 3.8 (-0.5), p<0.01<br>• 3.3 (-0.4), p<0.01
	Plans for future use of certain marketing instruments	The participants planned to use marketing instruments more rarely (statistically significant for 5/6 marketing instruments).<br>In general<br>• Drug sample<br>• Industry-sponsored continuing medical education<br>• One-on-one interactions with industry representatives<br>• Gifts less than CAN $10	Averages on a 5-pt.-Likert-scale (5=at every possible opportunity, 1=never)<br>n.s., p<0.01<br>• 3.1 (-0.5, p<0.01)<br>• 2.7 (-0.5, p<0.01)<br>• 2.2 (-0.3, p<0.01)<br>•<br>• 2.7 (-0.6, p<0.01)
	Use of certain marketing instruments in the past month	No statistically significant differences	• -
	Self confidence in identifying and managing different marketing instruments	No statistically significant differences	• -
**Stanley et al. 2005 [33]**	Knowledge about the pharmaceutical industry	Participants had a better score after the intervention compared to before	Average percentage of correct answers with standard error: 56.8% +/-3.3 after the intervention vs. 32.9% +/-3.7 before

	Attitudes toward the pharmaceutical industry	Participants showed a more positive attitude toward pharmaceutical companies in 2/6 items.<br><br>• The pharmaceutical industry overcharges the National Health Service.<br>• Pharmaceutical company bosses are 'fat cats'.	Average agreement on an 11-point-Likert-scale (0-10, 10= strong agreement) **<br><br>• ca. 5.5 (ca. -1, p<0.05)<br>• ca. 5.5 (ca. -1, p<0.05)
**Wofford & Ohl 2005 [29]**	Attitudes toward interactions with PSRs	For 2/4 items, participants showed a more positive attitude toward PSRs after the intervention (statistically significant).<br>• Detailing of pharmaceutical representatives has educational value for practicing physicians.<br>• Detailing of pharmaceutical representatives has educational value for medical students.<br><br>For 1/4 items, there was a trend toward a more positive attitude after the intervention.<br>• Information provided by pharmaceutical representatives is biased.<br><br>For 1/4 items, participants showed a more skeptical attitude toward PSR after the intervention (statistically significant)<br>• Pharmaceutical representatives are influential with regard to physicians' prescribing habits	Proportion of participants that agreed with the statement<br>• 43.2% (+25.5%, p<0.0001)<br><br>• 40.5% (+18.4%, p=0.0007)<br><br>• 72.9% (-11.2%, p=0.065)<br><br>• 62.1% (+7.9%, p=0.004)
**Wall et al. 2013 [30]**	Attitudes toward interactions with PSRs	For 1/6 questions there was a statistically significant difference compared to before the intervention<br>• Counter-detailing helps me better understand the proper use of medications detailed by Pharm Reps.	Agreement on a 5-pt.-Likert-scale, 5=strongly agree<br>• 5 (+1, p<0.01)
**Tillmanns et al. 2007 [21]**	Self-assessment of knowledge regarding interactions with the pharmaceutical industry<br><br>Interest in the topic of interactions with the pharmaceutical industry	Participants rated their knowledge to be higher after the intervention<br><br>Participants were more interested in the topic after the intervention	11-pt. rating scale (0-10, 10= the most knowledge); ca. 7.5 (ca. +3.8, p=0.00)**<br><br>11-pt. rating scale (0-10, 10= the most interest); ca. 7.8 (ca. +0.8, p=0.02)**
**Only posttest**			
**Kelcher et al. 1998 [20]**	Evaluation of an interaction with a PSR<br><br>Evaluation of the intervention	In discussions, residents could name advantages and disadvantages as well as costs of the drugs. Residents and the faculty discussing with them felt better informed after the intervention.<br><br>• The participants felt better prepared for the interactions with PSRs and thought the course should continue to be offered.<br>• The participants thought that regular visits from PSRs are not important or of small importance.	• -<br><br>• 11/12 (92%)<br><br>• 6/12 (50%)

**Only pretest**			
**Palmisano & Edelstein 1980 [25]**	Appropriateness of a gift	• Before the intervention, 46% of participants thought it was inappropriate for a medical student to accept a gift with a value of 50$ from a pharmaceutical company	• -
* Where not otherwise specified, the result at posttest is reported with the absolute change compared to the pretest in parentheses			
** Results read from a graph or figure, no exact numbers were reported in the publication			
